# Technology-Based Interventions for Physical Activity and Sedentary Behaviour in Adults: A Scoping Review

**DOI:** 10.3390/jfmk11020217

**Published:** 2026-05-29

**Authors:** Mariasole Antonietta Guerriero, Vittoria Lettieri, Fiorenzo Moscatelli, Giovanni Messina, Marcellino Monda, Antonieta Messina, Nicola Mancini, Maria Ruberto, Rita Polito

**Affiliations:** 1 Department of Humanistic Studies, University of Foggia, 71121 Foggia, Italy; mariasole.guerriero@unifg.it; 2 Department of Education and Sport Sciences, Pegaso Telematic University, 80143 Naples, Italy; vittoria.lettieri@unipegaso.it (V.L.); fiorenzo.moscatelli@unipegaso.it (F.M.); nicola.mancini@unipegaso.it (N.M.); maria.ruberto@unipegaso.it (M.R.); 3 Unit of Dietetics and Sports Medicine, Section of Human Physiology, Department of Experimental Medicine, University of Campania Luigi Vanvitelli, 80138 Naples, Italy; giovanni.messina@unicampania.it (G.M.); marcellino.monda@unicampania.it (M.M.); 4 Department of Precision Medicine, University of Campania Luigi Vanvitelli, 80131 Naples, Italy; antonietta.messina@unicampania.it; 5 Department of Psychology and Health Sciences, Pegaso Telematic University, 80143 Naples, Italy

**Keywords:** digital health, physical activity, sedentary behavior, wearable devices, mHealth interventions

## Abstract

**Background**: Physical inactivity and sedentary behaviour are major public health concerns associated with an increased risk of non-communicable diseases, reduced quality of life, and substantial healthcare burden. In recent years, technology-based interventions, including wearable devices, mobile health applications, artificial intelligence-driven systems, and adaptive digital platforms, have been increasingly adopted to promote physical activity and reduce sedentary time in adult populations. However, the evidence remains fragmented across intervention types, behavioural targets, and population groups. The aim of this scoping review was to map the recent literature on digital interventions designed to promote active lifestyles in adults, with a specific focus on their reported impact on physical activity promotion and sedentary behaviour reduction. **Methods**: This scoping review was conducted in accordance with the PRISMA-ScR guidelines. A literature search was performed in PubMed and Scopus using a predefined search strategy combining terms related to digital technologies, physical activity, sedentary behaviour, and adult populations. Studies published in English between 2022 and 2026 were considered. After removal of duplicates and screening of titles and abstracts, full texts were assessed according to predefined eligibility criteria. Data were charted descriptively and synthesised narratively to identify the main intervention models and emerging research trends. **Results**: The search identified 887 records, of which 35 studies were included in the final synthesis. The literature included was grouped into four broad categories: wearable devices and mHealth tools for monitoring and goal-setting; adaptive interventions based on Just-In-Time Adaptive Interventions, artificial intelligence, and gamification; advanced technologies such as Internet of Things systems and exoskeleton-based approaches; and hybrid interventions combining digital tools with human support or environmental modifications. Overall, technology-based interventions were generally associated with increases in step count, moderate-to-vigorous physical activity, and adherence to movement-related behaviours. In contrast, their effectiveness in reducing sedentary behaviour was less consistent and appeared to depend more strongly on context-sensitive prompting, posture-focused strategies, and multicomponent or hybrid intervention models. **Conclusions**: Digital health interventions represent a promising strategy for promoting physical activity in adults, but their impact on sedentary behaviour reduction remains more limited and heterogeneous. The findings suggest that simply increasing exercise is not sufficient to address prolonged sitting and that more tailored, adaptive, and context-aware approaches are needed. Future research should prioritise methodological standardisation, longer follow-up periods, and interventions specifically designed to interrupt sedentary time across different adult populations.

## 1. Introduction

Physical inactivity and prolonged sedentary behaviour represent one of the most pressing global public health challenges. According to the latest data, almost a third of the world’s adult population (31.3%, equivalent to around 1.8 billion people) is classified as physically inactive, an alarmingly worsening trend that is likely to exceed 35% by 2030 [1]. At a continental level, the situation remains critical: 45% of adults in the European Union report that they do not engage in any form of physical exercise or sport [2]. Physical inactivity and sedentary behaviour are related but distinct movement behaviours. Physical inactivity refers to not achieving the recommended levels of moderate-to-vigorous physical activity, whereas sedentary behaviour is defined as any waking behaviour characterized by an energy expenditure ≤ 1.5 metabolic equivalents while in a sitting, reclining, or lying posture. Although these behaviours frequently coexist, they represent different physiological and behavioural constructs and may require different intervention strategies. Both are recognised as major determinants of chronic non-communicable diseases and reduced quality of life [3].

As detailed by Bull et al. (2020) [4], adults are recommended to perform 150–300 min of moderate-intensity aerobic physical activity or 75–150 min of vigorous-intensity aerobic physical activity per week, together with muscle-strengthening activities involving major muscle groups on two or more days per week. Older adults are additionally encouraged to engage in multicomponent activities that emphasize balance and functional strength. Importantly, the guidelines also recommend limiting sedentary time and replacing it with physical activity whenever possible. Crucially, these updated guidelines introduce a specific recommendation to ‘limit the amount of time spent being sedentary’ and to ‘aim to replace sedentary time with physical activity of any intensity,’ acknowledging for the first time that every move counts toward mitigating the health risks of an inactive lifestyle. This distinction is crucial to understanding the emergence of the ‘Active Couch Potato’ phenomenon. As characterized by Owen et al. (2010) [5], this term identifies individuals who successfully meet standard physical activity recommendations—such as the 150 min weekly threshold—yet spend most of their remaining waking hours in sedentary postures. Clinical evidence suggests that the physiological risks associated with prolonged sitting, particularly regarding glucose metabolism and lipid profiles, persist independently of the time dedicated to structured exercise. Consequently, an individual can be simultaneously ‘physically active’ by guideline standards and ‘highly sedentary’ in their daily routine, a paradox that highlights the need for interventions capable of addressing both ends of the movement spectrum.

From this perspective, the population of young adults and university students takes on particular significance; as the future adult generation, they are at a critical transitional stage during which sedentary lifestyles and high levels of physical and mental stress can become entrenched, negatively affecting their long-term health and psychological well-being [6]. However, the issue extends throughout adulthood, with the workplace being one of the main catalysts. Entering the workforce and the office routine encourage the accumulation of hours of uninterrupted inactivity, with direct consequences for employees’ cardiometabolic risk, productivity and quality of life [7,8,9].

To address this issue, the World Health Organisation has published the Global Strategy on Digital Health 2020–2025, encouraging the integration of digital technologies (mHealth) and wearable devices to enhance prevention and promote self-management of health in a scalable and cost-effective manner [10,11,12]. Moving beyond the old paradigm of simple quantitative monitoring (such as the isolated ‘10,000 steps’ target), current guidelines and research are shifting towards a holistic management of ‘24 h behaviours’, which includes increased physical activity, posture monitoring and sleep hygiene [6,13]. To adhere to the WHO’s recommendation to break up sedentary behaviour consistently, the focus has shifted to innovative strategies such as ‘Snacktivity’ (short, frequent active breaks), aimed at breaking up daily inactivity in an acceptable way [14,15].

Technological innovation has also enabled the development of increasingly sophisticated interventions that integrate real-time ecological assessments (EMA) [16], artificial intelligence algorithms [11] and real-time adaptive systems (Just-In-Time Adaptive Interventions—JITAI), capable of providing personalised support based on the user’s actual context [17].

Given the rapid pace of this digital evolution and the clinical need to distinguish between the effectiveness of promoting physical activity and that of reducing sedentary behaviour, a systematic mapping of the evidence is required. Therefore, the aim of this scoping review is to analyse scientific literature to assess the effectiveness of digital interventions in promoting an active lifestyle among adults, identifying emerging technologies and remaining research gaps.

## 2. Materials and Methods

**Figure 1:**
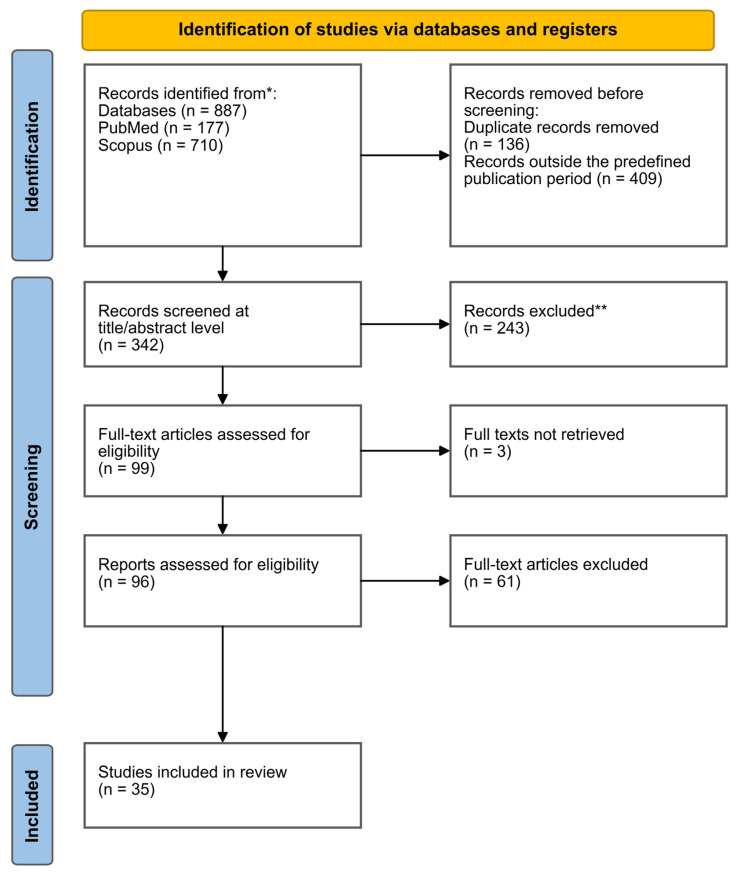
PRISMA flow diagram of the study selection process. A total of 887 records were identified through database searching (PubMed, *n* = 177; Scopus, *n* = 710). After removal of 136 duplicate records, 751 records remained. Of these, 409 records were excluded because they fell outside the predefined publication period (2022–2026), leaving 342 records for title and abstract screening. Following screening, 243 records were excluded. Ninety-nine full-text articles were assessed for eligibility; three full texts could not be retrieved, resulting in 96 reports assessed in detail. After full-text evaluation, 61 articles were excluded because they did not meet the eligibility criteria or did not adequately address the review objectives. Ultimately, 35 studies were included in the final synthesis. * record identified included; ** record excluded.

This scoping review was conducted in accordance with the Preferred Reporting Items for Systematic Reviews and Meta-Analyses extension for Scoping Reviews (PRISMA-ScR) guidelines [18]. The PRISMA-ScR framework was used to guide the identification, screening, eligibility assessment, and final inclusion of the studies. The PRISMA flow diagram summarizing the study selection process is presented in Figure 1, and the completed PRISMA-ScR checklist is provided in the Supplementary Materials.

The aim of this review was to map the recent scientific literature on technology-based interventions designed to promote physical activity and/or reduce sedentary behaviour in adults, with particular attention to the type of digital technology employed, the characteristics of the intervention, and the main behavioural outcomes reported.

### 2.1. Information Sources and Search Strategy

The literature search was conducted in two major bibliographic databases, PubMed and Scopus, selected for their broad coverage of biomedical, behavioural, and health technology research. The search strategy was designed to identify studies investigating digital or technology-based interventions targeting physical activity and sedentary behaviour in adult populations.

The following search string was used:

(“artificial intelligence” OR “machine learning” OR wearable* OR “fitness tracker” OR smartwatch* OR pedometer* OR accelerometer* OR “activity monitor”) AND (“physical activity” OR exercise OR fitness OR “physical exercise”) AND (adult* AND (sedentary OR inactive OR “physically inactive”)).

The search strategy was designed to maximize sensitivity for studies involving digital technologies, physical activity, and sedentary behaviour. Terms such as “intervention” and “program” were not included in the search string because many technology-based studies describe behavioural support using alternative terminology (e.g., digital health, mHealth, coaching, prompting, monitoring, adaptive systems). The aim was therefore to avoid unnecessarily restricting the retrieval of potentially relevant evidence. Nevertheless, this decision may have reduced the sensitivity of the search for some intervention-focused studies and is acknowledged as a limitation.

The search terms were applied to the title and abstract fields to ensure the retrieval of studies with a clear focus on the topic of interest. The search was restricted to studies published between 2022 and 2026 in order to capture the most recent evidence in a rapidly evolving field. Only articles published in English were considered. Preprints were excluded. All records identified through the database search were exported and managed using Zotero reference management software, version 9.0.3, which was used to organize citations and remove duplicate records. The initial search yielded 887 records in total, of which 177 were retrieved from PubMed and 710 from Scopus. After duplicate removal, 751 unique records remained for further screening (Figure 1). No protocol registration was performed for this scoping review. The completed PRISMA-ScR checklist is provided as Supplementary Materials.

### 2.2. Eligibility Criteria

Studies were selected according to predefined eligibility criteria based on population, intervention characteristics, outcomes, and study design.

Regarding the population, only studies involving adult human participants aged 18 years or older were considered eligible. Studies focusing exclusively on paediatric or adolescent populations were excluded. Studies conducted in clinical or pathological populations were considered eligible only when the intervention specifically targeted physical activity promotion and/or sedentary behaviour reduction as primary behavioural aims.

Regarding the intervention, studies investigating technology-based interventions or providing relevant evidence on their effectiveness were included. Eligible technologies included, but were not limited to, wearable devices, fitness trackers, smartwatches, pedometers, accelerometers, mobile health applications, artificial intelligence-based systems, machine learning-supported interventions, ecological momentary interventions, and other technology-assisted behavioural tools. Studies focused exclusively on technical validation, device accuracy, sensor performance, or methodological testing were excluded. However, in line with the evidence-mapping purpose of a scoping review, observational studies, secondary analyses, systematic reviews, and study protocols were retained when they provided relevant information regarding technology use, behavioural monitoring, adherence patterns, intervention architecture, or contextual determinants of physical activity and sedentary behaviour.

With respect to outcomes, studies were included if they examined physical activity promotion and/or sedentary behaviour reduction as primary outcomes of the intervention. Studies in which physical activity was reported only as a secondary endpoint, or in which the primary focus was unrelated to behavioural movement outcomes, were excluded.

As for study design, all empirical quantitative, qualitative, and mixed-methods designs were considered eligible if they investigated a structured intervention. Review articles, protocols, and other secondary sources were considered only when relevant to the mapping objective and if consistent with the scoping nature of the review. Preprints were excluded. In line with the exploratory nature of scoping reviews, both primary empirical studies and secondary sources (e.g., systematic reviews, meta-analyses, and study protocols) were considered, provided they contributed to mapping the characteristics and scope of technology-based interventions targeting physical activity and sedentary behaviour.

### 2.3. Study Selection Process

The study selection process was conducted in three sequential stages: identification, screening, and inclusion.

#### 2.3.1. Identification Phase

During the identification phase, 887 records were retrieved from the database search. After exportation to Zotero, 136 duplicate records were identified and removed, resulting in 751 unique articles. Subsequently, 409 records were excluded because they fell outside the predefined publication period of 2022 to 2026. This left 342 studies eligible for title and abstract screening.

#### 2.3.2. Screening Phase

In the screening phase, the titles and abstracts of the 342 remaining records were independently assessed by two reviewers. Studies were screened according to the predefined eligibility criteria concerning target population, intervention characteristics, outcomes, and study design. Discrepancies between reviewers were resolved through discussion until consensus was reached.

At this stage, studies were excluded if they focused on non-adult populations, did not include a relevant digital or technology-based component, addressed only technical device validation or sensor accuracy, or did not consider physical activity promotion or sedentary behaviour reduction as a relevant behavioural outcome. Observational studies and secondary sources were retained only when they contributed meaningfully to the evidence-mapping objective of the review. The reasons for exclusion at the title and abstract stage are summarized in Table 1.

Following this screening process, 243 records were excluded and 99 articles were retained for full-text assessment.

#### 2.3.3. Inclusion Phase

In the inclusion phase, the full texts of the 99 selected studies were retrieved and examined in detail by the reviewers. Three studies were excluded because the full text could not be retrieved for detailed assessment. A further 61 articles were excluded after full-text evaluation because they did not fully meet the eligibility criteria or did not adequately address the research question.

More specifically, studies were excluded at this stage if they were conducted in clinical populations without physical activity promotion or sedentary behaviour reduction as the primary behavioural focus, if they did not include a genuine technology-based intervention, or if physical activity and sedentary behaviour were peripheral rather than central outcomes of the study. No restrictions were applied with regard to the specific type of technology employed, provided that the digital component constituted a central element of the intervention.

At the end of the full-text review, 35 studies were included in the final synthesis.

### 2.4. Data Charting and Extraction

Data from the included studies were charted using a structured extraction approach developed for this review. For each study, the following information was extracted: author and year of publication, study design, characteristics of the population, sample size, intervention type, technological device or platform used, intervention duration, and main outcomes related to physical activity and/or sedentary behaviour.

The data extraction process was performed to ensure a consistent and descriptive mapping of the available evidence. Extracted information was then organized into summary tables to facilitate comparison across studies and to support the identification of recurring intervention models, target populations, and behavioural outcomes.

### 2.5. Data Synthesis

As expected in a scoping review, the purpose of the synthesis was not to provide a pooled quantitative estimate of effect, but rather to map the breadth, characteristics, and emerging patterns of the literature. Therefore, a descriptive and narrative synthesis was conducted.

Following full-text review and data charting, the included studies were grouped into broad thematic categories according to their main technological architecture and intervention logic. This process led to the identification of four overarching categories: (1) wearable devices and mHealth interventions for monitoring and goal-setting; (2) adaptive interventions based on Just-In-Time Adaptive Interventions (JITAI), artificial intelligence, and gamification; (3) advanced technologies, including Internet of Things systems and exoskeleton-based interventions; and (4) hybrid and multicomponent approaches combining digital tools with human support, environmental modifications, or clinical supervision.

This thematic organization was adopted to provide a structured overview of the field and to highlight similarities and differences across intervention models, behavioural targets, and implementation contexts.

## 3. Results

Beyond their technological architecture, the included studies can also be interpreted according to three behavioural targets: interventions primarily designed to increase physical activity, interventions specifically intended to reduce sedentary behaviour, and hybrid approaches aiming to integrate movement into daily life through combinations of monitoring, adaptive prompting, environmental modification, and human support. This distinction is important because increasing physical activity and reducing sedentary behaviour are related but not interchangeable objectives.

### 3.1. Wearable Devices and mHealth for Monitoring and Goal-Setting

A substantial body of research is based on the combined use of commercial activity trackers (e.g., Fitbit, Garmin) and smartphone apps or web platforms, designed for continuous monitoring and goal-setting. Meta-analyses and umbrella reviews suggest that these devices are generally associated with increases in daily steps and time spent in physical activity, whereas their impact on sedentary behaviour appears limited or inconsistent [19,20,21,22]. Monitoring and structured feedback also appear to support increases in steps and muscle strength among community-dwelling older adults [23,24], favourable cardiometabolic profiles when movement and posture are objectively assessed through thigh accelerometry [25], and higher step counts among patients undergoing clinical weight management [26]. It has also been observed that long-term use of these platforms generates specific trajectories of improvement, with only a fraction of users (approximately 17%) responding with immediate peaks in activity [27]. Obese women in a workplace setting have benefited from the use of accelerometers (Movband) with both step-based protocols and short structured exercise sessions [28]. The uptake and usability of these tools are generally good [29], although sustained use requires tailored metrics and seamless integration into daily routines to prevent users from dropping out [30].

### 3.2. Adaptive Interventions (JITAI), Artificial Intelligence and Gamification

A significant step forward from simple monitoring is represented by technologies that send personalised prompts in real time. Artificial intelligence approaches, including clustering algorithms such as k-means, may help identify behavioural phenotypes and patterns of adherence to tool use in mHealth cardiac rehabilitation programmes [31]. In older adults, the use of ecological momentary prompts (EMPs) resulted in an increase of over 5500 steps per week and an improvement in executive functions [32]. At the same time, the integration of gamification elements within apps (e.g., Sweatcoin) was associated with more favourable outcomes compared with the use of a pedometer alone, including reductions in fatigue and improvements in step count and sleep quality among haemodialysis patients [33].

### 3.3. Advanced Technologies: The Internet of Things (IoT) and Exoskeletons

One area of research focuses on technologies that directly alter the environment or the body’s mechanics. In offices, IoT systems (such as smart cups with LEDs to remind users to take breaks) have improved the automatic nature of behaviour, without, however, drastically reducing the total number of minutes spent sitting [34]. On the other hand, wearable robotics, using modular hip exoskeletons, has been associated with meaningful improvements in walking function, reduced muscular effort, and reductions in sedentary time in both older adults and young adults [35,36].

### 3.4. Hybrid Support (Tech + Touch) and Multicomponent Interventions in Clinical Populations

Several of the more promising interventions targeting populations with specific health conditions or vulnerabilities combined digital tools with human support or environmental components. In cancer survivors, the addition of telephone coaching or social media support groups significantly increased MVPA [37] or reduced sedentary behaviour [38], although the isolated use of a smartwatch during hospitalisation did not show clear benefits for physical activity [39]. Social support may also moderate intervention effects, as older adults with weaker offline social networks appeared to benefit more from the virtual support provided by web-based interventions [40]. Hybrid interventions have successfully reduced sedentary behaviour in post-stroke patients [41] and in individuals with type 2 diabetes through the RESIT programme [42,43]. In pregnant women, a combined intervention (tracker, standing desk, coaching) reduced sedentary time by replacing it with standing, without increasing step count [44]. The human factor, combined with wearable devices for pacing and monitoring, also appeared to support improvements in psychological outcomes and movement-related behaviours among patients with chronic pain [45,46,47] and in primary care patients with depression at one-year follow-up [48]. Multi-component interventions (PF-Life) and future large-scale trials confirm the trend towards platforms that combine structured clinical exercise and mobile health [49,50,51] (Table 2).

## 4. Discussion

This scoping review mapped recent evidence on technology-based interventions designed to promote physical activity and reduce sedentary behaviour in adults. Overall, digital technologies appear promising for supporting increases in step count, moderate-to-vigorous physical activity, and adherence to movement-related behaviours. However, the findings also indicate that reducing sedentary behaviour requires a distinct intervention architecture. While physical activity promotion often relies on goal setting, self-monitoring, and feedback mechanisms, sedentary behaviour reduction depends more strongly on context-sensitive prompts, posture monitoring, environmental restructuring, and behavioural interruption strategies. An important observation emerging from the literature is that sedentary behaviour should not be considered merely the inverse of physical activity. Individuals may achieve recommended levels of exercise while simultaneously accumulating prolonged periods of sitting, a phenomenon commonly described as the “Active Couch Potato” paradigm. Consequently, interventions designed to increase exercise participation may not automatically reduce sedentary time. This distinction may explain why wearable activity trackers consistently improve step count and physical activity outcomes, whereas their impact on sedentary behaviour remains more variable and context-dependent.

Firstly, the evidence suggests a clear gap in effectiveness across outcomes. Meta-analyses and reviews [19,20,21], supported by longitudinal studies [22,23,24,26], suggest that commercial devices appear effective in supporting movement promotion through visual feedback and goal setting. Nevertheless, standard monitoring-only technologies appear less effective at interrupting prolonged periods of sitting, particularly in workplace or home environments [34]. To address sedentary behaviour more effectively, real-time adaptive interventions may represent particularly relevant strategies, including JITAI systems [32,52,53], as well as approaches that shift the focus from step counting to the accurate assessment of posture and sitting patterns [25]. Modifying the environment (e.g., adjustable desks) and encouraging regular interruptions of sitting time appears to be a pragmatic and potentially effective strategy in populations with mobility limitations, fatigue, or clinical impairments, such as pregnant women [44] and post-stroke patients [41].

Secondly, the literature reviewed challenges the paradigm of the ‘universal device’ or one-size-fits-all intervention [51]. Age may also act as an important moderator of intervention effectiveness. A considerable proportion of the included studies involved older adults, who may present different levels of digital literacy, technology acceptance, functional capacity, and support needs compared with younger adults. Therefore, future research should investigate not only the effectiveness of specific technologies, but also how age-related factors influence engagement, adherence, and behavioural outcomes. Physiological and behavioural responses to app use are fragmented into highly subjective trajectories [27]. Rigid technologies may contribute to fatigue or dropout if they are not integrated with motivational components such as gamification [33], AI-based personalization algorithms [31], or hardware specifically designed to support users’ physical needs, as in the case of exoskeletons [35,36]. In workplace programmes, effectiveness declines if not supported by an organisational culture that allows for autonomy of movement [28]. Long-term use (sustained use) absolutely requires that the device adapts to the user, reducing interface friction [30].

Finally, the concept of “Tech + Touch” emerges as one of the most relevant findings of this review. Digital technologies should not necessarily be viewed as fully autonomous solutions capable of generating behavioural change independently. Rather, the evidence suggests that the most promising interventions combine technological functions—such as monitoring, self-monitoring, real-time feedback, adaptive prompting, and personalization—with human elements including coaching, social support, professional guidance, and accountability [37,38,40,42,43,46,49,50]. Human behaviour is not only the target of these interventions but also an active component of the intervention process itself. Several studies included in this review demonstrated that technology-supported interventions achieved greater engagement and more favourable behavioural outcomes when accompanied by telephone counselling, social support groups, physiotherapist supervision, health coaching, or structured educational components [37,38,41,42,43,46,48]. This pattern was particularly evident in clinical and vulnerable populations, where digital tools alone often appeared insufficient to sustain long-term behaviour change [39,45,47]. Interestingly, the benefits of hybrid approaches were also amplified among individuals with lower levels of offline social support, suggesting that technology may act as a facilitator of social connectedness rather than merely a behavioural monitoring tool [40]. Consequently, future digital health interventions may benefit from hybrid models in which technology enhances behavioural support rather than replacing it. From this perspective, artificial intelligence, Just-In-Time Adaptive Interventions (JITAIs), and wearable technologies should be considered complementary components within broader behavioural ecosystems that integrate technological innovation with human interaction, contextual adaptation, and long-term motivational support [32,52,53].

### 4.1. Bias Assessment and Study Limitations

This study has several limitations that should be considered when interpreting the findings. First, no formal methodological quality appraisal or risk-of-bias assessment was conducted, in accordance with the objectives and methodological framework of a scoping review. Consequently, the present review should not be interpreted as providing definitive evidence of clinical efficacy or as a basis for clinical recommendations. Rather, it offers a descriptive and interpretative mapping of recent literature, highlighting promising technological approaches, emerging trends, and research gaps.

A second limitation concerns the literature search strategy. Although we searched two of the most comprehensive and authoritative databases in the biomedical and technological fields (PubMed and Scopus), the decision not to include further specific databases may have limited the identification of additional relevant studies. Furthermore, the search strategy did not explicitly include terms such as “intervention” or “program”. Although this decision was intended to maximize sensitivity for diverse technology-based approaches, it may have reduced the identification of some intervention-focused studies. Similarly, the emphasis on sedentary and inactive behaviours may have favoured the retrieval of studies targeting risk reduction rather than broader health-promotion strategies. Furthermore, the decision to include only articles in English published within a narrow timeframe (2022–2026) may have introduced a selection bias, a priori excluding valid research conducted in different geographical contexts or technologies that laid the foundations for the sector in the preceding years. Furthermore, the strict application of eligibility criteria led to the exclusion of a significant number of studies that focused exclusively on the technical validation of devices (e.g., sensor accuracy) or in which increased physical activity was merely a secondary outcome. Although this decision was useful to maintain a precise focus on behavioural outcomes and technology-based intervention models, it may have partially narrowed the scope of the results, omitting preliminary data on emerging technologies.

Finally, there is marked heterogeneity among the included studies in terms of the devices used, the duration of the interventions and, above all, the assessment metrics (e.g., daily steps vs. minutes of moderate-to-vigorous activity vs. objectively measured or self-reported sedentary time). This methodological heterogeneity makes it difficult to directly compare the effectiveness of the various digital protocols.

### 4.2. Implications and Future Directions

Considering the findings and the methodological limitations highlighted, several priority areas for future research have emerged. Firstly, there is a clear need to conduct long-term longitudinal studies (with follow-up periods exceeding 6–12 months). Much of the current literature focuses on short-term outcomes; to understand the true clinical efficacy of digital technologies, it will be essential to assess the maintenance of behavioural change over time, overcoming the initial physiological decline in adherence often associated with the adoption of a new device.

Secondly, as current technologies appear more consistently associated with increases in physical activity volume than with reductions in uninterrupted sedentary behaviour, future studies should focus on developing protocols aimed exclusively at interrupting time spent sitting. It will be crucial to optimise adaptive systems (JITAI) and artificial intelligence algorithms to deliver contextual prompts that promote active micro-breaks (Snacktivity), especially in work environments, whilst minimising the risk of ‘alert fatigue’ for the user. Finally, we need more standardisation to overcome the current fragmentation of literature. Future research should prioritise repeatable protocols, using the same wearable devices across different populations and settings. In addition, researchers must adopt shared methods for analysing wearable data. The focus should shift from simple step counting to a holistic approach, mapping movement and posture over a full 24 h period.

## 5. Conclusions

This scoping review highlights the growing potential of digital technologies to support movement-related behaviours in adults. Overall, the evidence suggests that wearable devices, mobile health applications, adaptive digital platforms, and artificial intelligence-based systems may contribute to increases in physical activity, particularly through self-monitoring, goal setting, personalized feedback, and behavioural reinforcement strategies [19,20,21,22,23,24,26,29,30]. However, the findings also indicate that promoting physical activity and reducing sedentary behaviour should not be considered interchangeable objectives. While technology-based interventions consistently appear capable of increasing step count and moderate-to-vigorous physical activity, their effectiveness in reducing prolonged sedentary time remains more heterogeneous and context-dependent [21,25,32,34,41,42,43,44].

A central finding emerging from this review is that sedentary behaviour reduction may require a distinct intervention architecture. Unlike physical activity promotion, which can often be supported through monitoring and feedback mechanisms, reducing sedentary behaviour appears to depend more strongly on context-aware prompting, posture-sensitive monitoring, environmental modifications, and adaptive behavioural strategies capable of interrupting prolonged sitting throughout the day [25,32,34,41,44]. This distinction reinforces the importance of moving beyond a purely exercise-centred perspective toward a broader understanding of 24 h movement behaviours.

The review further highlights the relevance of hybrid “Tech + Touch” approaches. The most promising interventions were not necessarily those relying exclusively on technological sophistication, but rather those integrating digital tools with coaching, social support, professional guidance, behavioural counselling, or structured human interaction [37,38,40,42,43,46,48,49,50]. In this context, technology appears most effective when used to enhance behavioural support rather than replace it. Human involvement remains essential for fostering motivation, accountability, contextual adaptation, and long-term adherence, particularly among older adults and individuals with chronic health conditions [39,40,45,47].

Finally, future research should prioritize methodological standardisation, longer follow-up periods, and greater consistency in outcome assessment across studies. Particular attention should be paid to the development of adaptive and context-sensitive interventions, including Just-In-Time Adaptive Interventions (JITAIs) and artificial intelligence-driven systems, while simultaneously investigating how individual characteristics such as age, digital literacy, social support, and clinical status influence engagement and behavioural outcomes [31,32,52,53]. Advancing this integrated perspective will be essential for developing more effective and sustainable digital health strategies capable of simultaneously promoting physical activity and reducing sedentary behaviour across diverse adult populations.

## Figures and Tables

**Table 1:** Inclusion and exclusion criteria in the screening phase at the abstract and title level.

Category	Inclusion Criteria	Exclusion Criteria
Target Population	Adult human beings (≥18 years).	Pediatric populations (<18 years); clinical/pathological populations (unless the intervention is specifically aimed at physical activity as the primary outcome).
Intervention Type	Structured interventions based on the use of digital technologies (e.g., mHealth, artificial intelligence, wearables, fitness trackers) as a core element.	Absence of a relevant digital or technology-based component; studies focused exclusively on technical validation, device accuracy, sensor performance, or methodological testing without contributing to the mapping of technology-based strategies for physical activity promotion or sedentary behaviour reduction.
Outcome	Promotion of physical activity and/or reduction in sedentary behavior as the primary outcome of the study.	Technical validation or monitoring of devices (e.g., sensor accuracy); physical activity is assessed only as a secondary parameter.
Study Design	Empirical quantitative, qualitative, mixed-methods studies, structured trials, secondary analyses, systematic reviews, meta-analyses, and protocols when relevant to the evidence-mapping objective of the scoping review.	Pre-print studies.
Search Parameters	Publication between 2022 and 2026; English language articles.	Publications prior to 2022; languages other than English; Full-text not available for eligibility assessment.

**Table 2:** Features of the included results.

Author, Year	Study Design	Population (*N*, Age, Target)	Intervention (Device & Duration)	Main Outcomes
Adachi et al. (2025) [27]	Retrospective observational study	Physically inactive adult males with CV risk factors (*N* = 1369, median age 55)	Mystar app + Fitbit Inspire. Lifestyle modification program (goal setting, feedback, professional chat) for 6 months.	3 step-count trajectories identified; 17% significantly increased steps in the first 3 months; higher app usage correlated with more PA.
Aguilar-Latorre et al. (2023) [48]	Multicenter open-label RCT	Adults ≥ 18 years with depression in primary care (*N* = 188, mean age 53)	Smartwatch for tracking steps/sleep + Lifestyle Modification Programme (6 weekly 90 min sessions).	Intervention significantly reduced depressive symptoms and sedentariness compared to treatment as usual.
Ahmadi et al. (2024) [25]	Cross-sectional IPD meta-analysis	12,095 adults (mean age 54.5)	Thigh accelerometers (ActivPAL, Axivity, ActiGraph). Observational free-living measurement (no intervention).	64 min/day walking and 5 min/day stairs associated with a favorable cardiometabolic profile; >12.1 h/day sedentary time associated with unfavorable profile.
Alley et al. (2023) [29]	Process evaluation of RCT	Inactive adults ≥ 65 years (*N* = 174)	Fitbit Flex 1 + computer-tailored web platform (Active for Life) for 12 weeks.	Good engagement in both groups; usability was modest but higher in the Fitbit group; participants appreciated motivation and accountability.
Alley et al. (2025) [40]	Secondary analysis of RCT	Inactive adults ≥ 65 years (*N* = 243, mean age 69)	Fitbit + computer-tailored web platform (Active for Life) for 12 weeks.	In participants with low social support, the intervention increased MVPA significantly more than control; no effect in high social support group.
Balkić Widmann et al. (2023) [45]	Secondary analysis of RCT	Adults 18–80 years with chronic low back pain (*N* = 41)	Xiaomi Mi Fit Band 2. Interdisciplinary chronic pain management program for 4 weeks (+4 weeks follow-up).	Steps and active minutes increased (non-significant); significant improvements in anxiety, depression, stress, and catastrophizing.
Barua et al. (2025) [31]	Secondary analysis of RCT	Adults ≥ 65 years with ischemic heart disease (*N* = 271)	Fitbit Inspire + tablet + app. mHealth-CR program (aerobic exercise, remote monitoring, counseling) for 3 months.	High adherence to accelerometer weakly associated with 6-MWD improvement; AI clusters (k-means) identify behavioral phenotypes.
Biddle et al. (2026) [43]	Mixed-methods process evaluation of feasibility RCT	Adults 18–85 years with Type 2 Diabetes (*N* = 70)	Wearables (Xiaomi, Garmin, zTrack) + apps/software. RESIT program (online education, coaching, self-monitoring) for 6 months.	82% completed online education; 73% tech usage at 6 months; wearables perceived as very useful for reducing sitting.
Brierley et al. (2024) [42]	RCT feasibility trial (mixed methods)	Adults 18–85 years with Type 2 Diabetes (*N* = 70)	Wearable devices + smartphone app + computer prompt software (RESIT intervention) for 3 months.	Sitting reduction of −30.9 min/day (intervention) vs. −4.4 min/day (control) at 3 months; improved quality of life and self-efficacy.
Bronas et al. (2024) [32]	Pilot RCT	Latino community-dwelling adults, 55–89 years (*N* = 39)	Fitbit Charge 4 + SMS EMI (ecological momentary interventions) via app for 6 weeks.	Steps/week increased (+5543); sedentary time decreased (−348 min); executive function (TMT-B) improved significantly. High compliance (79%).
Cebrick-Grossman & Fetherman (2024) [28]	Pretest-posttest RCT	Sedentary obese female workers/nurses (*N* = 12)	Movband (wrist-worn accelerometer). 12-week workplace intervention: HIIT group vs. STEP group.	Significant reduction in body measurements. HIIT group: increased lean mass. STEP group: increased daily steps.
Christensen et al. (2025) [50]	RCT Protocol (phase 3)	Adults with CKD (eGFR 20–60), ≥20 years	ActivPAL + behavioral coaching (SLIMM) to reduce sedentariness + guided resistance training.	Study protocol in progress; no results available yet.
Chua et al. (2024) [22]	Systematic review & meta-regression	Adults with Type 2 Diabetes (19 RCTs, *N* = 2547)	Wearable technologies (pedometers, accelerometers, smartwatches).	Wearables increased daily steps by 1583/day and reduced systolic blood pressure by 2.46 mmHg; very low certainty of evidence.
Dennett et al. (2025) [39]	Single-blind feasibility RCT	Hospitalized adult cancer survivors (*N* = 24)	Fitbit Inspire + activPAL + step diary. 2 behavioral sessions during admission.	Safe and acceptable, but no clear advantage on PA; mobility showed a moderate effect for the intervention at discharge.
Ding et al. (2026) [23]	Pragmatic longitudinal single-arm intervention	Community-dwelling older adults ≥ 50 years (*N* = 4344)	Smart bracelet + mobile app + smart dynamometer. 12-month hybrid program (monitoring, feedback, sessions).	MVPA increased over time; higher MVPA trajectories were associated with greater grip strength gains.
Elzeky et al. (2025) [33]	Single-blind quasi-experimental study	Adults on hemodialysis, 18–<60 years (*N* = 90)	Smartphone app (Fitbit + Sweatcoin) vs. pedometer vs. control (12 weeks).	Gamified group improved steps, fatigue, and sleep quality vs. pedometer and control; no effect on physical function.
Fanning et al. (2022) [46]	Randomized pilot trial	Older adults (55–85 years) with obesity and chronic pain (*N* = 44)	iPad, Fitbit, wireless scale, mHealth app, Zoom coaching. 12-week group-mediated intervention.	Large effects for steps and postural changes; small effect on reducing prolonged sedentary bouts; pain improved in both groups.
Firkin et al. (2026) [52]	Study protocol	Planned 20 physically inactive adults	Apple Watch + iPhone app + JITAI server architecture. Planned 6-week intervention.	Protocol only; behavioral and feasibility outcomes not yet reported.
Gibbs et al. (2024) [44]	Pilot/feasibility RCT (2:1)	Adults in first trimester of pregnancy (*N* = 51)	Fitbit/Apple Watch + activPAL + stand-up desk + Facebook group + remote coaching (SPRING intervention).	Intervention feasible and acceptable; sedentary time reduced (−0.84 h/day) and standing time increased (+0.77 h/day); no significant increase in steps.
Grunberg et al. (2022) [47]	Secondary analysis/single-arm observational	Adults with chronic pain (*N* = 41)	Fitbit + ActiGraph. 10-week mind–body program with step and pacing goals.	Significant increase in steps over time; reaching Fitbit goals was associated with improved anxiety and physical function.
Hardcastle et al. (2024) [37]	RCT	Breast/colorectal cancer survivors in remote areas (*N* = 87)	Fitbit Charge 2 + telephone coaching for 12 weeks.	Net improvement in MVPA (+49.8 min/week); no difference between groups for sedentary behavior.
Hendrickx et al. (2024) [41]	Randomized multiple baseline study	Highly sedentary post-stroke individuals (*N* = 14)	RISE eCoaching system, activity monitor, app, PT support for 15 weeks.	Significant reduction in sedentary time (~1.3 h/day) and increased fragmentation; better results with participatory support.
Herring et al. (2025) [26]	Service evaluation/real-world pre-post	Adults with severe obesity (*N* = 559 eligible, 290 registered)	Steps4Health mHealth tool + optional wearable linkage.	High registration rate; clinically relevant mean increase of 974 steps/day in the first 6 weeks.
Ho et al. (2024) [24]	Clustered trial	Community-dwelling older adults > 60 years (*N* = 58)	Asus VivoWatch BP + smartphone app. 8-week intervention with progressive targets (5000 to 7500 steps/day) + nutrition education.	Total and light PA increased; skeletal muscle index (SMI) and muscle strength increased; sedentary time, BMI, and waist circumference decreased vs. control.
Huang et al. (2023) [34]	Mixed methods feasibility study, single-group pre-post	Adult office workers (*N* = 15)	IoT system WorkMyWay: wearable + Android app + LED smart cup reminder. 6-week intervention to interrupt occupational sedentariness.	Study feasible and acceptable; no significant change in OSPA, but improved automaticity and memory of breaks.
Jayaraman et al. (2022) [35]	Pre-post intervention study	Community-dwelling older adults > 65 years (*N* = 12)	Modular hip exoskeleton GEMS-H + ActiGraph. 12 community gait training sessions (30 min each) with exoskeleton.	Clinically significant improvements in balance, gait speed, and endurance; significant reduction in sedentary time.
Johnson et al. (2022) [38]	Pilot RCT	Young adult cancer survivors 18–39 years (*N* = 50)	Fitbit + Facebook group + text messaging + buddy system (12 weeks).	Intervention feasible; significant reduction in sedentary time compared to control, but no difference for MVPA.
Koh et al. (2025) [53]	Scoping review	Adults with excess body weight (35 studies)	JITAI, mobile apps, wearables, EMA/passive sensing.	Described JITAI on diet, PA, and self-weighing; reported improvements in weight, PA, and sedentariness.
Larsen et al. (2022) [19]	Systematic review & meta-analysis	Adults 18–65 years (121 RCTs, *N* = 16,743)	Physical activity monitors (PAMs) with feedback.	Moderate effect on physical activity (~1235 steps/day), small effect on MVPA (~48.5 min/week), non-significant effect on sedentary time.
Lee et al. (2024) [36]	RCT (3 parallel groups)	Healthy young adults 19–65 years (*N* = 45)	Wearable hip exoskeleton Bot Fit + Samsung Galaxy Watch 4 + mobile app. 18 sessions of walking exercise in 6 weeks.	Improved muscle strength, reduced muscle effort, improved pelvic tilt symmetry; higher exercise volume/intensity (steps, distance, energy, HR).
Lewis et al. (2025) [51]	Review/narrative update	Adults (synthesis of literature)	mHealth, smartphones, trackers, chatbots, gamification, smart home.	Summarized advances in interventions for sedentariness and mHealth; described recent trends and future directions.
Li et al. (2024) [49]	Prospective single-cohort mixed-methods feasibility pilot	Community-dwelling pre-frail older adults ≥ 65 years (*N* = 16)	PF-Life mHealth platform: web portal + smartphone app + wearable ActiGraph. 8-week lifestyle-integrated multicomponent program.	High feasibility/usability; improvements in HGS, TUG, and chair rise; sedentary behavior decreased (−33 min/day) and low PA increased (+31 min/day).
Li et al. (2025) [20]	Systematic review & meta-analysis	Community-dwelling older adults (23 RCTs, *N* = 4566)	Wearable activity trackers (pedometers, Fitbit, Jawbone, Garmin, etc.).	Increase in physical activity time and daily step count vs. usual care; no significant improvement in sedentary time, body composition, or physical function.
Longhini et al. (2024) [21]	Umbrella review	Adults (51 systematic reviews included)	Wearable devices/activity monitors.	Possible increase in steps/day and MVPA; inconsistent results for sedentary behavior.
Manskow et al. (2023) [30]	RCT, mixed-methods	Inactive adults at risk of lifestyle diseases (*N* = 183)	Mi Smart Band 5 + PAI app + online training + Facebook social support. 18-month intervention.	PAI showed better adoption/usability; social support had high acceptability; online training had low adoption; results focused on sustained use.

## Data Availability

No new data were created or analyzed in this study. Data sharing is not applicable to this article.

## Supplementary Materials

The following supporting information can be downloaded at: https://www.mdpi.com/article/10.3390/jfmk11020217/s1, Table S1. PRISMA-ScR Checklist.
