# Supplementary material for: Technology-Based Interventions for Physical Activity and Sedentary Behaviour in Adults: A Scoping Review
Source: J Funct Morphol Kinesiol. 2026 May 29;11(2):217. doi: 10.3390/jfmk11020217 (PMC13302658; doi:10.3390/jfmk11020217)
Supplement: Supplementary file 1 [file jfmk-11-00217-s001.zip › jfmk-4287445-supplementary.pdf]

# Table S1. PRISMA-ScR Checklist

*Technology-Based Interventions for Physical Activity and Sedentary Behaviour in Adults: A Scoping Review*

**Note:** This checklist was prepared according to the PRISMA Extension for Scoping Reviews (PRISMA-ScR). Page numbers refer to the submitted manuscript version.

**Protocol registration:** No protocol registration was performed for this scoping review.

| Section | Item | PRISMA-ScR Item | Checklist Recommendation | Reported | Location | Comments |
|---|---|---|---|---|---|---|
| TITLE | 1 | Title | Identify the report as a scoping review. | Yes | Page 1 | The title clearly identifies the manuscript as a scoping review. |
| ABSTRACT | 2 | Structured summary | Provide a structured summary that includes background, objectives, eligibility criteria, sources of evidence, charting methods, results, and conclusions that relate to the review questions and objectives. | Yes | Page 2 | The abstract includes Background/Objectives, Methods, Results, and Conclusions. |
| INTRODUCTION | 3 | Rationale | Describe the rationale for the review in the context of what is already known. Explain why the review questions/objectives lend themselves to a scoping review approach. | Yes | Pages 3–4 | The Introduction explains the public health relevance of physical inactivity and sedentary behaviour, the role of digital technologies, and the need to map recent evidence. |
| INTRODUCTION | 4 | Objectives | Provide an explicit statement of the questions and objectives being addressed with reference to their key elements. | Yes | Page 4 | The review objective is clearly stated at the end of the Introduction. |
| METHODS | 5 | Protocol and registration | Indicate whether a review protocol exists; state if and where it can be accessed; and if available, provide registration information. | Not registered | Methods section / Not applicable | No protocol registration was performed for this scoping review. This should be stated in the Methods section or in the cover response to the editor. |
| METHODS | 6 | Eligibility criteria | Specify characteristics of the sources of evidence used as eligibility criteria and provide a rationale. | Yes | Pages 6–8; Table 1 | Eligibility criteria are described according to population, intervention characteristics, outcomes, study design, and search parameters. |
| METHODS | 7 | Information sources | Describe all information sources in the search and the date the most recent search was executed. | Partially reported | Pages 4–5 | PubMed and Scopus are reported as databases. The manuscript reports the publication period 2022–2026. If available, the exact date of the last search should be added. |
| METHODS | 8 | Search | Present the full electronic search strategy for at least one database, including any limits used. | Yes | Page 5 | The full search string and applied limits are reported. |
| METHODS | 9 | Selection of sources of evidence | State the process for selecting sources of evidence included in the scoping review. | Yes | Pages 7–9; Figure 1 | The manuscript describes identification, screening, and inclusion phases. |
| METHODS | 10 | Data charting process | Describe the methods of charting data from the included sources of evidence. | Yes | Page 9 | The Data Charting and Extraction section describes the extraction process. |
| METHODS | 11 | Data items | List and define all variables for which data were sought and any assumptions or simplifications made. | Yes | Page 9 | Extracted variables include author/year, design, population, sample size, intervention type, device/platform, duration, and main outcomes. |
| METHODS | 12 | Critical appraisal of individual sources of evidence | If done, provide rationale for conducting critical appraisal and describe the methods used. | Not applicable | Page 20; Section 4.1 | No formal risk-of-bias or methodological quality assessment was conducted, consistent with the scoping review design. This is |

| | | | | | | |
|---|---|---|---|---|---|---|
| | | | | | | explicitly acknowledged in the Limitations section. |
| METHODS | 13 | Synthesis of results | Describe the methods of handling and summarizing the data that were charted. | Yes | Page 9 | The manuscript reports descriptive and narrative synthesis organized into thematic categories. |
| RESULTS | 14 | Selection of sources of evidence | Give numbers of sources screened, assessed for eligibility, and included, with reasons for exclusions at each stage. | Yes | Pages 6–9; Figure 1 | The PRISMA flow diagram reports 887 records identified, 99 full texts assessed, and 35 studies included. |
| RESULTS | 15 | Characteristics of sources of evidence | For each source of evidence, present characteristics for which data were charted and provide citations. | Yes | Table 2; Pages 11–17 | Table 2 reports author/year, design, population, intervention/device and duration, and main outcomes. |
| RESULTS | 16 | Critical appraisal within sources of evidence | If done, present data on critical appraisal of included sources of evidence. | Not applicable | Page 20; Section 4.1 | No formal critical appraisal was conducted. |
| RESULTS | 17 | Results of individual sources of evidence | For each included source of evidence, present the relevant data that were charted. | Yes | Table 2; Pages 11–17 | The main outcomes of each included source are summarized in Table 2. |
| RESULTS | 18 | Synthesis of results | Summarize and/or present the charting results as they relate to the review questions and objectives. | Yes | Pages 9–17 | Results are synthesized according to four technological/intervention categories and three behavioural targets. |
| DISCUSSION | 19 | Summary of evidence | Summarize the main results, including an overview of concepts, themes, and types of evidence available. | Yes | Pages 17–20 | The Discussion summarizes the evidence around physical activity promotion, sedentary behaviour reduction, context-aware interventions, and Tech + Touch models. |
| DISCUSSION | 20 | Limitations | Discuss the limitations of the scoping review process. | Yes | Pages 20–21 | The manuscript discusses the absence of risk-of-bias assessment, search strategy limitations, language/time restrictions, and heterogeneity across studies. |
| DISCUSSION | 21 | Conclusions | Provide a general interpretation of the results with respect to the review questions and objectives, as well as potential implications and/or next steps. | Yes | Pages 21–22 | The Conclusions summarize implications for digital health, adaptive interventions, sedentary behaviour reduction, and future research. |
| FUNDING | 22 | Funding | Describe sources of funding for the included sources of evidence and the scoping review, and describe the role of the funders. | Yes | Funding section | The manuscript states that the research received no external funding. |

**Editorial note:** The completed PRISMA flow diagram is included in the main manuscript as Figure 1. The checklist may be submitted as supplementary material.
